# Comparative Stepwise Pattern of Reactive Oxygen Species Production during *In Vitro* Development of Fertilized and Nuclear Transferred Goat Embryos

**DOI:** 10.22074/ijfs.2017.5049

**Published:** 2017-02-16

**Authors:** Mehdi Hajian, Sayed Morteza Hosseini, Somayyeh Ostadhosseini, Mohammad Hossein Nasr-Esfahani

**Affiliations:** 1 Department of Reproductive Biotechnology, Reproductive Biomedicine Research Center, Royan Institute for Biotechnology, ACECR, Isfahan, Iran; 2 Department of Embryology, Reproductive Biomedicine Research Center, Royan Institute for Reproductive Biomedicine, ACECR, Tehran, Iran

**Keywords:** Somatic Cell Nuclear Transfer, Reactive Oxygen Species, Goat

## Abstract

**Background:**

A unique feature of embryo metabolism is production of reactive oxygen species (ROS). It is well established that during *in vitro* culture, ROS levels increase over normal ranges observed for embryos developed *in vivo*. This study evaluates and compares the stepwise pattern of ROS production during *in vitro* development of reconstructed goat embryos produced by zona-free method of somatic cell nuclear transfer (SCNT). Furthermore, the pattern of ROS production of SCNT embryos were compared with zona free embryos derived from *in vitro* fertilization (IVF).

**Materials and Methods:**

In this experimental study, zona-free oocytes, SCNT and IVF embryos at different stages of *in vitro* development (2, 4, 8, 16-cells, morula, and blastocyst) were used for assessment of ROS production using 2, 7-dichloro dihydroflourescein diacetate (DCHFDA) probe and the result were presented as fold increase or decrease relative zona free oocytes.

**Results:**

The relative level of ROS compared to metaphase-II (MII) oocytes insignificantly decrease during early stages post embryo reconstitution and regained its value by 8-cell and morula stage and, significantly increase compared to MII oocytes by blastocyst stage.

**Conclusion:**

The pattern of ROS change in SCNT embryos is similar to zona free IVF derived embryos, except it decrease from two cell stage and regain its value at morula stage. The sudden rise in ROS at blastocyst stage, further emphasizes the special need of IVF and SCNT derived embryos during this stage of development.

## Introduction

The efficiency of blastocyst derived from *in vitro* embryo production (IVP) is around 30-40%, (1) and the birth rate per embryos transferred is around 35- 45% in domestic animals (2), despite great advances achieved in this field, during the past two decades. These rates of *in vitro* and *in vivo* developmental competence are almost invariably lower for embryos produced by somatic cell nuclear transfer (SCNT) technique, probably because, SCNT embryos have altered gene expression and metabolism due to improper epigenetic reprogramming (3).

Through optimized of zona free SCNT procedure in goat, we were able to reach a cloning efficiency (live birth) of 28.6% per transfer or 6.9% per embryo transfer and live birth of 21.42% per transfer or 5.2% per embryo transfer (4) which is substantially higher than those previously reported in the literature for goat (5, 6). Despite high cloning efficiency obtain in this approach; we believe there is still room to further improvement. One approach is to increase reprogramming efficiency at epigenetic level (7) and an alternative approach is to improve the intervening techniques in zona free SCNT like oocyte maturation, activation protocols, single versus group culture, and culture condition (8).

Literature study reveal that across all the species studied, including goat, composition of embryo culture media have profound effects on *in vitro* and *in vivo* embryo development (8). In regard to this, a characteristic feature of embryos produced *in vitro*, is high production of reactive oxygen species (ROS) (9, 10) and goat is not of any exception, which to our knowledge has not been so far studied. During *in vitro* embryo culture, ROS level increases in a cell cycle dependent manner compared to the *in vivo* embryos at similar stages (9, 11). SCNT is a more complicated process in which oocyte is exposed to various media and high degrees of *in vitro* manipulation which may adversely affect oxidation-reduction (REDOX) state of developing embryo, a situation so called oxidative stress (11). Therefore, it is important to understand how different method of embryo production [*in vitro* fertilization (IVF vs. SCNT)] can effect ROS generation.

The association between increased generations of ROS with zygote genomic activation (ZGA), also known as maternal to embryo transition, was first reported by Nasr-Esfahani and Johnson (9) in mice as an *in vitro* effect which it is well established that early embryonic block, and the rise in generation of ROS during IVP are also maternally derived and are independent of paternal contribution (10). During SCNT, maternal chromosome is totally replaced with diploid nuclei of a somatic cell without any contribution by sperm. Therefore, it is interesting to know, how the absence of maternal and paternal chromosomes and presence of somatic cell nuclei affect pattern of ROS generation in developing SCNT embryos. Such information may reveal light on how cytoplasm may regulate production of ROS and may also help investigators to understand if, and what extent, antioxidant compounds could be used to improve the efficiency of SCNT reconstructed embryos. In farm animals, Dalvit et al. (12) and Ostad Hosseini et al. (13) studied patterns of ROS production during *in vitro* development of cattle and sheep embryos, respectively. But, there is no study on pattern of ROS production during *in vitro* development of goat IVF or SCNT embryos. Therefore, the aim of this study was to develop and compare patterns of ROS production during different stages of pre-implantation of zona free IVF and zona free SCNT embryos in goat.

## Materials and Methods

This study was approved by the Ethical Committee of Royan Institute. In this experimental study, unless otherwise stated, all chemicals and media used in the present study were obtained from Sigma (St. Louis, MO, USA) and Gibco (Life Technologies, Rockville, MD, USA), respectively.

### Oocyte in vitro maturation

Abattoir-derived ovaries were used for oocyte *in vitro* maturation (IVM) as described previously (4). In brief, cumulus oocyte complexes (COCs) were aspirated from antral follicles and cultured in maturation medium comprised of tissue culture medium199 (TCM199) supplemented with 10% fetal calf serum (FCS), Na-pyruvate (2.5 mM), L-glutamine (1 mM), penicillin (100 IU/mL), streptomycin (100 mg/mL), follicle-stimulating hormone (FSH, 10 mg/mL), luteinizing hormone (LH, 10 mg/mL), estradiol-17β (1 mg/mL), cysteamine (0.1 mM), epidermal growth factor (EGF, 100 ng/mL) plus insulin-like growth factor (IGF, 100 ng/mL) for 20-22 hours under mineral oil at 38.5°C, 5% CO_2_, and maximum humidity.

### Donor cell preparation

Ear biopsy of a healthy pre-pubertal female goat was taken, cut into 2-3 mm^2^ fragments and cultured as explants in Dulbecco’s Modified Eagle Medium F-12 (DMEM/F-12) containing 10% FCS and antibiotic (1% penicillin-streptomycin) at 37°C, 5% CO_2_ in air. Cell started to shed out of the explants. Eventually, these cells proliferate to forms a confluent monolayer within 2-3 weeks. Obtained cells were used for investigation of fibroblast phenotype using differential immunostaining with anti-vimentin (for fibroblasts) and pan-cytokeratin (for epithelial cells) antibodies (7). Confirmed fibroblasts at passages 3-5 were used for SCNT experiments. In order to provide a synchronized population of G0/G1, cells were first cultured at 2.5×104 cells/cm^2^, and at the next day, the cells were washed thrice with phosphate buffer saline (PBS) before being cultured in medium that contained 0.5% FCS for 4-5 days. Serum starved cells were subsequently trypsinized and used for SCNT procedure.

### Somatic cell nuclear transfer procedure

*In vitro* matured oocytes were denuded by vortexing in presence of 300 IU/mL hyalorunidase. Only good quality oocytes with homogenous cytoplasm and extruded first polar body were used for the experiments. The process of zona free enucleation was carried out as described previously by Nasr-Esfahani et al. (4). In brief, zona was removed by brief enzymatic digestion [5 mg pronase in 1 mL of Hepes-TCM199 (HTCM) for 1 minute] followed by incubation in TCM199 free of pronase and containing 20% FCS to neutralize the remaining enzyme. It has been demonstrated that goat matured oocytes revealed a cytoplasmic extrusion cone which is clearly visible upon zona removal (14). This extrusion is considered as a hallmark of MII spindle during enucleation. The cytoplasmic extrusion was gently aspirated into a 2-3 μm pipette and with a gentle touch against the blind needle (5-10 μm), the MII extrusion was separated from the oocyte. Successful enucleation was confirmed by staining the separated MII extrusion with H33342 (5 μg/ml, 5 minutes). During this procedure enucleated oocyte are not exposed to UV.

Nuclear transfer (NT) was carried out according to Hosseini et al. (15). In brief, individual fibroblasts were adhered to oocytes in medium containing 10 mg/ml phytohemagglutinin. Subsequently, the couplets were electrofused in 290 mOsm fusion buffer [0.3 M Mannitol, 100 μM MgSO_4_, 50 μM CaCl2, 500 μM hepes, 0.05% bovine serum albumin (BSA)]. The reconstructed oocytes were rested for 0.5 hours before being activated using ionomycin (5 μM, 1 minut) followed by incubation with 2 mM 6-DMAP for 2 hours. Reconstituted-activated oocytes were then cultured in groups of five to seven in modified synthetic oviductal fluid (mSOF) under mineral oil at 38.5°C, 5% CO_2_, 5% O_2_ and humidified air for 7 days in 20 μl wells.

### In vitro fertilization procedure

According to Forouzanfar et al. (16), matured COCs were washed in fertilization medium and groups of 20-25 COCs were transferred into 100 μl droplets of fertilization medium under mineral oil. Five straws of frozen spermatozoa were thawed at 37ºC for 1 minute, pooled and washed through Pure Sperm (Nidacon, Gothenburg, Sweden) gradient (40 and 80%) to separate the motile spermatozoa from the immotile by centrifugation [700 g for 15 minutes at room temperature (RT)]. Matured COCs were inseminated with a final concentration of two million sperm per ml. The inseminated COCs were incubated for 22 hours in 5% CO_2_ in humidified air at 38.5°C. Twenty-two hours after insemination, cumulus cells attached to oocytes were mechanically removed via pipetting. Then, the zona was removed by brief enzymatic digestion as described above. The presumptive zygotes were then cultured in groups of five to seven as described for SCNT embryos.

### Reactive oxygen species measurement

The process of ROS measurement was as described previously (13). In brief, stock solutions of 2, 7-dichloro dihydroflourescein diacetate (DCHFDA, Sigma D6883, 5 mM) were prepared in dimethyl sulfoxide (DMSO) and stored at -20°C in dark. For each experiment, 5 μM working solution were prepared by dilution in TCM199 containing 1 mg/ mL poly vinyl alcohol (PVA). To measure ROS levels, 15-30 embryos per replicate derived from zona free IVF or SCNT were pooled from different stages of embryo development (2-cells, 4-cells, 5-8-cells, and greater than 8-cells, morula and expanded blastocysts). Zona free metaphase-II (MII) oocytes were also simultaneously assessed. Samples were incubated with 5 μM of DCFHDA in TCM199 for 30 minutes in incubator. Samples were then washed in TCM199, placed in 5 μl droplets covered by mineral oil and then immediately exposed to UV light of a fluorescent microscope (Olympus, IX71, Japan) and observed using filter sets (excitation wavelength: 450-490 nm, emission wavelength: 515-565 nm). Digital images of individual oocyte or embryo were taken with a high sensitive camera (DP-72, Olympus, Japan). Background, positive and negative controls were taken to account for fluorescence or inter-experimental variations. Fluorescent intensity of each taken image was assessed by Image J (National Institute of Mental Health, Bethesda, MD, USA). To reduce variations and possible errors, when comparison between different groups was required, experiments were designed so that oocytes and embryos from each group were available for assessment at the same period. To further minimize inter-experimental variation, the relative fluorescence intensity of each embryonic stage to the mean intensity of MII-oocytes in the same experiment was calculated according to the below formula:

The relative intensity is defined as the difference in the intensity of embryos from the mean intensity of MII-oocytes/mean intensity of MII-oocytes. It is important to note that for assessment of ROS at each embryonic stage at least three replicates were carried out. For each replicate at least 30 to 80 embryos and 55 to 95 oocytes were assessed.

### Statistical analysis

Percentages data were transformed by ArcSin and analyzed by one way ANOVA model of SPSS version 17 (SPSS, Science, Chicago, IL, USA). Differences were compared by the Tukey multiple comparison post hoc test. All data are expressed as mean ± SEM and differences were considered as significant at P<0.05.

## Results

### Reactive oxygen species measurement

**Fig.1:**
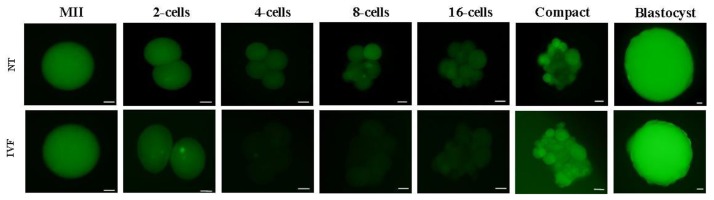
Representative fluorescence images of goat zona free MII-oocyte, IVF and SCNT-derived embryos at different stages of preimplantation embryo development. Oocytes were stain with DCFHDA for ROS measurement. Bar represents 25 μm. MII; Metaphase-II, IVF; *In vitro* fertilization, SCNT; Somatic cell nuclear transfer, DCFHDA; 2, 7-dichloro dihydroflourescein diacetate, ROS; Reactive oxygen species, and NT; Nuclear transfer.

Figure 1 shows fluorescence images of goat MII-oocyte, zona free SCNT and IVF embryos at different stages of pre-implantation embryo development following staining with DCFHDA for ROS measurement. As depicted, irrespective of embryo production method, fluorescence intensity increased as the embryo progressed toward blastocyst stage.

**Fig.2:**
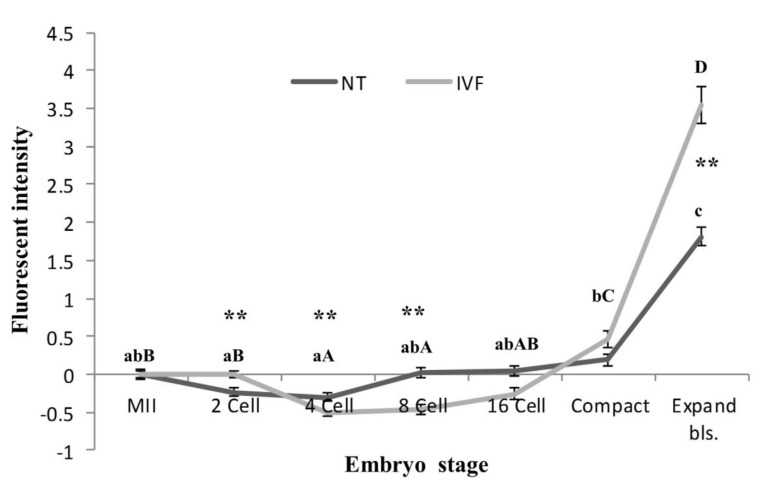
Comparative analysis of the mean ROS level of MII oocytes, zona free IVF and SCNT embryos assessed using the DCHFDA probe. ROS; Reactive oxygen species, MII; Metaphase-II, IVF; *In vitro* fertilization, SCNT; Somatic cell nuclear transfer, DCFHDA; 2, 7-dichloro dihydroflourescein diacetate, NT; Nuclear transfer, a-c; Different letters showed significant differences within the NT derived embryos, A-D; Different letters showed significant differences within the IVF derived embryo, **; In each embryo stage showed significant differences between NT and IVF groups.

Figure 2 shows the mean relative intensities of embryos relative to MII-oocytes at different stages of development in zona free IVF and SCNT derived embryos. As shown, in zona free IVF embryos, the mean relative ROS levels significantly decreased at 4 and 8- cell stages relative to the mean intensity of MII-oocytes and then began to rise by 16 cell stage which resulted in a significant increases by compact and blastocyst stages relative to all the earlier stages. Moreover, the relative increase at the blastocyst stage was significantly higher than compact embryos.

The trend of ROS production in SCNT embryos appear to follow the same trend as those of zona free IVF embryos. In zona free IVF embryos the decrement in ROS occur after 2-cell stage while in zona free SCNT reconstructs, the decrements being at earlier stage (post reconstruction). Subsequently, the increment in ROS production in zona free IVF embryos begin at around 8-cell stage while the increment in zona free SCNT embryo begin at around 4-cell stage. Therefore, despite similar trend of ROS production, a significant difference between the two groups were observed at 2- (lower in SCNT group), 4- (higher in SCNT group) and 8- (higher in SCNT group) cell stages. A significant difference was also observed at blastocyst stage. The degree of ROS production was significantly higher in blastocyst derived from zona free IVF embryos in comparison to zona free SCNT reconstructs.

**Fig.3:**
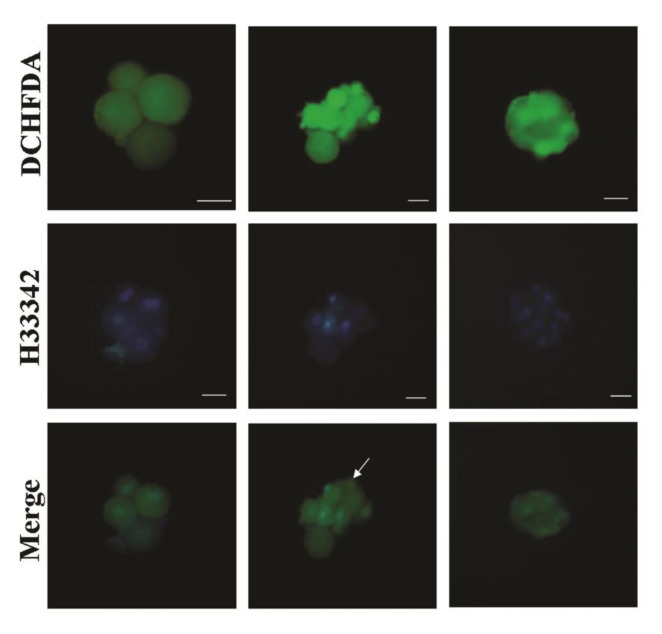
Confirmation of presence of nuclei in cloned embryos. During the SCNT procdure, some embryos may become fragmented and providing blastomeres without nuclei. Therefore, combined staining with nuclear dye (H33342) and DCHFDA was carried out to investigate this phenomenon. As show, except for one blastomere without nucleus (arrow), all the other blastomeres with high intensity for DCHFDA had nuclei. Bar represents 50 μm. SCNT; Somatic cell nuclear transfer and DCFHDA; 2, 7-dichloro dihydroflourescein diacetate.

During this study, unlike the zona free IVF embryos, in some of the SCNT embryos one or more blastomeres showed higher fluorescence intensity compared to other blastomeres (Fig .3).

Also, it has been reported that some blastomeres, due to asymmetrical division, are non-nucleated. In order to understand if this phenomenon has any relation to intensive ROS levels within blastomeres, the SCNT embryos with non-uniformed ROS staining were also stained with viable chromatin dye (H33342, 5 μg/ml for 5 minutes) and no relation was observed between ROS intensity with presence or absence of chromatin in each blastomeres. As shown in Figure 3, except for one blastomere without nucleus (arrow), all the other blastomeres with high ROS intensity had nuclei.

## Discussion

The results of this study showed that the relative ROS production in zona free IVF derived embryos decreased following fertilization, began to rise at around zygote genomic transition (ZGA) which occur around 8-16 cell stage (17), and substantially increased from compaction to the blastocyst stage. The overall trend of ROS pattern in developing SCNT embryos was similar to zona free IVF embryos, except for the time of ROS raise that apparently took place at earlier stages (4-8 cell stage) in SCNT embryos. The increase in relative ROS production around the ZGA in consistent with the previous reports in other species [mice: Nasr-Esfahani and Johnson (9)], [bovine: Dalvit et al. (12)], [sheep: Ostad Hosseini et al. (13)]. During ZGA maternal stores of RNA become gradually depletion and embryo begins to rely on its own genome transcription. The earlier raise of ROS in SCNT embryos might be related to difference in mRNA clearance, mitochondrial activation, or the depletion of antioxidant capacity (like glutathione (GSH) content) during SCNT or genomic reprogramming which needs further investigations (18).

Although, the pattern of ROS production is species-specific, it has been established that the stage of ZGA in developing embryos of mice and other animals investigated so far, coincides with a sharp increase in ROS level (19, 20). In this regard, a number of studies have shown that antioxidant supplementation of culture medium, particularly around the peak of ROS production, improves developmental competence of embryos (13, 21-23), thus suggesting a link between REDOX state and ZGA arrest embryos. In agreement, it is frequently reported that when first embryonic division commences, majority of the cleaved embryos may progressed to the stage which coincides with ZGA irrespective of their initial quality. Therefore, arrest around the ZGA period is considered the bottleneck of *in vitro* embryo development (18). In this sense, this study for the first time in the goats show that the increase in ROS production also occurs around the ZGA stage, and therefore, supplementation of antioxidant around 8 to 16 -cells stages and after that when ROS level substantially increases, may improve *in vitro* development of goat embryos.

In accordance with previous reports in other species, (9, 12, 13), we also observed a substantial raise in ROS at compaction and blastocyst stage in both groups. This rise in ROS production, is very likely to be related to a switch from anaerobic to aerobic glycolysis, since the ATP production becomes dependent on Krebs cycle after ZGA while before this stage ATP production is mainly dependent on glycolysis. The degree of ROS production was significantly higher in IVF compared to SCNT derived blastocysts and this is likely due to higher quality and metabolic activity of IVF derived embryos, but this conclusion needs further validation. Another interesting observation in this study was the higher ROS production in some nucleated blastomeres of SCNT embryos which was rarely seen in IVF derived embryos. The reason for this difference remains to be elucidated.

## Conclusion

This study for the first time described the pattern of ROS production in reconstructed embryo derived from SCNT procedure and in zona free goat embryo. The results showed two major time points of increased ROS production. The first raise in ROS production was observed during ZGA and the second raise took place during the period of blastocyst formation. These results may emphasize the special need of SCNT and zona free IVF derived embryos to external source of antioxidants during these two critical stages of development which in turn may affect the efficiency of embryo production from these two techniques.
